# Exploring innovative strides in radiolabeled nanoparticle progress for multimodality cancer imaging and theranostic applications

**DOI:** 10.1186/s40644-024-00762-z

**Published:** 2024-09-20

**Authors:** Atena Najdian, Davood Beiki, Milad Abbasi, Ali Gholamrezanezhad, Hojjat Ahmadzadehfar, Ali Mohammad Amani, Mehdi Shafiee Ardestani, Majid Assadi

**Affiliations:** 1 The Persian Gulf Nuclear Medicine Research Center, Bushehr Medical University Hospital, School of Medicine, Bushehr University of Medical Sciences, Bushehr, Iran; 2 Research Center for Nuclear Medicine, Tehran University of Medical Sciences, Tehran, Iran; 3 Department of Medical Nanotechnology, School of Advanced Medical Sciences and Technologies, Shiraz University of Medical Sciences, Shiraz, Iran; 4 Department of Radiology, Keck School of Medicine, University of Southern California (USC), 1441 Eastlake Ave Ste 2315, Los Angeles, CA 90089 USA; 5 Department of Nuclear Medicine, Klinikum Westfalen, Dortmund, Germany; 6 Department of Nuclear Medicine, Institute of Radiology, Neuroradiology and Nuclear Medicine, University Hospital Knappschaftskrankenhaus, Bochum, Germany; 7 Department of Radiopharmacy, Faculty of Pharmacy, Tehran University of Medical Sciences, Tehran, Iran

**Keywords:** Radiolabeled nanoparticles, Multimodality imaging, Theranostics, Targeting, Cancer

## Abstract

Multimodal imaging unfolds as an innovative approach that synergistically employs a spectrum of imaging techniques either simultaneously or sequentially. The integration of computed tomography (CT), magnetic resonance imaging (MRI), single-photon emission computed tomography (SPECT), positron emission tomography (PET), and optical imaging (OI) results in a comprehensive and complementary understanding of complex biological processes. This innovative approach combines the strengths of each method and overcoming their individual limitations. By harmoniously blending data from these modalities, it significantly improves the accuracy of cancer diagnosis and aids in treatment decision-making processes. Nanoparticles possess a high potential for facile functionalization with radioactive isotopes and a wide array of contrast agents. This strategic modification serves to augment signal amplification, significantly enhance image sensitivity, and elevate contrast indices. Such tailored nanoparticles constructs exhibit a promising avenue for advancing imaging modalities in both preclinical and clinical setting. Furthermore, nanoparticles function as a unified nanoplatform for the co-localization of imaging agents and therapeutic payloads, thereby optimizing the efficiency of cancer management strategies. Consequently, radiolabeled nanoparticles exhibit substantial potential in driving forward the realms of multimodal imaging and theranostic applications. This review discusses the potential applications of molecular imaging in cancer diagnosis, the utilization of nanotechnology-based radiolabeled materials in multimodal imaging and theranostic applications, as well as recent advancements in this field. It also highlights challenges including cytotoxicity and regulatory compliance, essential considerations for effective clinical translation of nanoradiopharmaceuticals in multimodal imaging and theranostic applications.

**Graphical Abstract:**

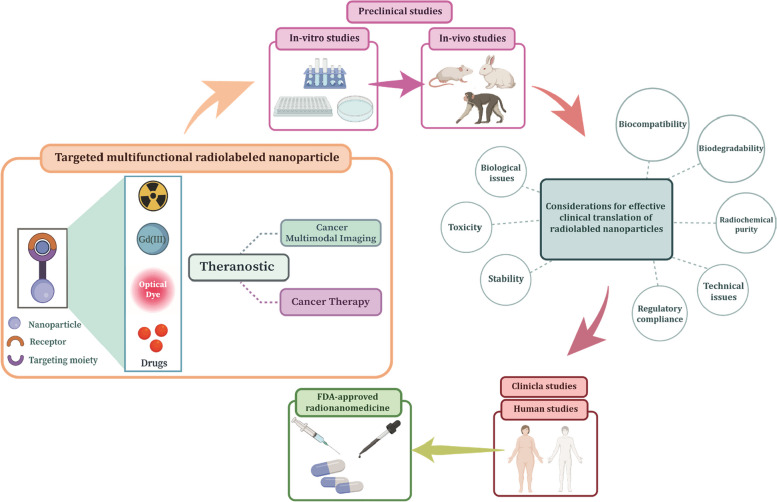

## Introduction

Cancer is a disease of continual, unregulated proliferation of cells, which may spread to distant organs in the body abruptly [1]. It continues to be the leading cause of death worldwide, despite the large number of studies and great advances seen during the past decade [2]. The coronavirus disease 2019 (COVID-19) pandemic has negatively affected the diagnosis and treatment of cancer in 2020 [3]. In fact, increased mortality was seen because of delays in diagnosis and treatment of cancers. Fear of COVID-19 exposure and reduced access to care because of health care setting closures were the most common causes of cancer deaths [4].

**Fig. 1:**
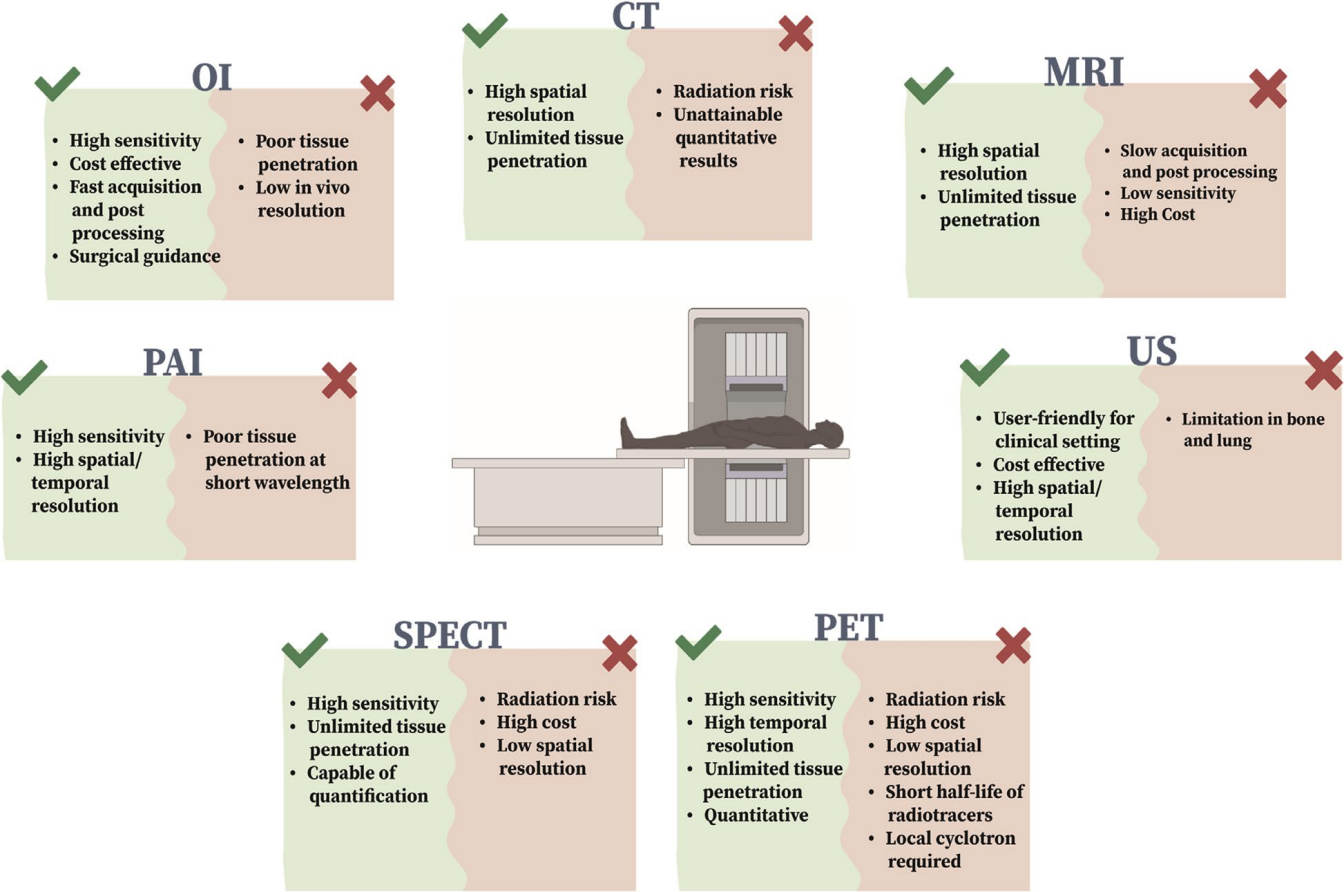
A schematic representation depicting various molecular imaging modalities is provided, along with their corresponding advantages and disadvantages

Since the early 1990s, molecular imaging has been developed as a non-invasive tool to visualize the biological functions and mechanisms of living organisms at molecular and cellular levels. Molecular imaging methods facilitate diagnosing the progress of different diseases, the biodistribution of drugs, in vivo molecular events, and evaluating metabolic processes in real time [5]. Various imaging techniques can be divided into two groups: anatomic/structural imaging and functional imaging. Anatomical imaging depicts the exact location and area of interest in the body, with particular properties related to the imaging method employed. On the contrary, functional imaging provides details on the molecular conditions of specific organs or tissues and represents the spatial distribution of physiological processes based on the particular imaging modality employed. Magnetic resonance imaging (MRI) with outstanding soft tissue contrast and x-ray computed tomography (CT) with high spatial resolution produce three-dimensional anatomic imaging without penetration depth concerns. Optical imaging (OI) and nuclear imaging (SPECT and PET) provide quantitative functional information on biological events at the molecular level [6–10]. Molecular imaging modalities have distinct advantages as well as inherent limitations (refer to Fig. 1). For instance, OI provides high sensitivity and fast data acquisition with low-cost contrast, yet it suffers from low penetration depth and high spatial resolution; MRI and CT have high spatial resolution but often achieve low sensitivity; SPECT and PET offer high sensitivity and strong penetration depth, but they have poor spatial resolution. Furthermore, clinical applications of CT, PET, and SPECT are limited due to the risks associated with ionizing radiation [11]. Table 1 summarizes the characteristics of each imaging modality.

**Table 1:** Characteristics of different molecular imaging modalities [12]

Imaging modality	Form of Energy Used	Temporal Resolution	Spatial Resolution	Sensitivity	Depth of Penetration	Safety Profile	Cost	Clinical Translation
Ultrasound (US)	ultrasound	Seconds-minutes	0.01–0.1 mm (few mm depth) 1–2 mm (few cm depth)	10^–12^ M	mm-cm	Good	Low	Yes
CT	X-rays	Minutes	50–200 µm (microCT) 0.5–1 mm (clinical)	Not determined	Limitless	Ionizing radiation	Medium	Yes
Optical fluorescence imaging (FI)	Visible to infrared light	Seconds-minutes	2–3 mm	10^–9^-10^–12^ M	< 1 cm	Good	Low–High	Yes
Optical bioluminescence Imaging (BLI)	Visible to infrared light	Seconds-minutes	3–5 mm	10^–15^-10^–17^ M	1–2 cm	Good	Low	No
MRI	Radio- frequency waves	Minutes-hours	0.01–0.1 mm (small-animal MRI) 0.5–1.5 mm (clinical)	10^–3^-10^–5^ M	Limitless	No ionizing radiation	High	Yes
SPECT	Gamma rays	Minutes	0.5–2 mm (microSPECT) 7–15 mm (clinical)	10^–10^-10^–11^ M	Limitless	Ionizing radiation	Medium–High	Yes
PET	Annihilation photos	Seconds-minutes	1–2 mm (microPET) 6–10 mm (clinical)	10^–11^-10^–12^ M	Limitless	Ionizing radiation	High	Yes
Photoacoustic imaging (PAI)	near-infrared light or radiofrequency-wave	Seconds-minutes	10 µm to 1 mm	Not determined	6 mm to 5 cm	Good	Low	Clinically Translatable

Multimodality imaging with two or more imaging techniques enables the combination of the strengths of individual modalities while overcoming their limitations. In particular, integration of structural and functional images with the utilization of incorporated single photon emission computed tomography/computed tomography (SPECT/CT) and positron emission tomography/computed tomography (PET/CT) has been indicated to be highly effective and useful [13–15]. A multimodal imaging approach ideally has the purpose of precisely visualizing the exact localization, metabolic activity of the target organ or tissue, and pathological mechanisms at the molecular level. Thus, it ensures enormous benefits for improving the diagnosis and therapeutic assessment of a disease [16].

**Fig. 2:**
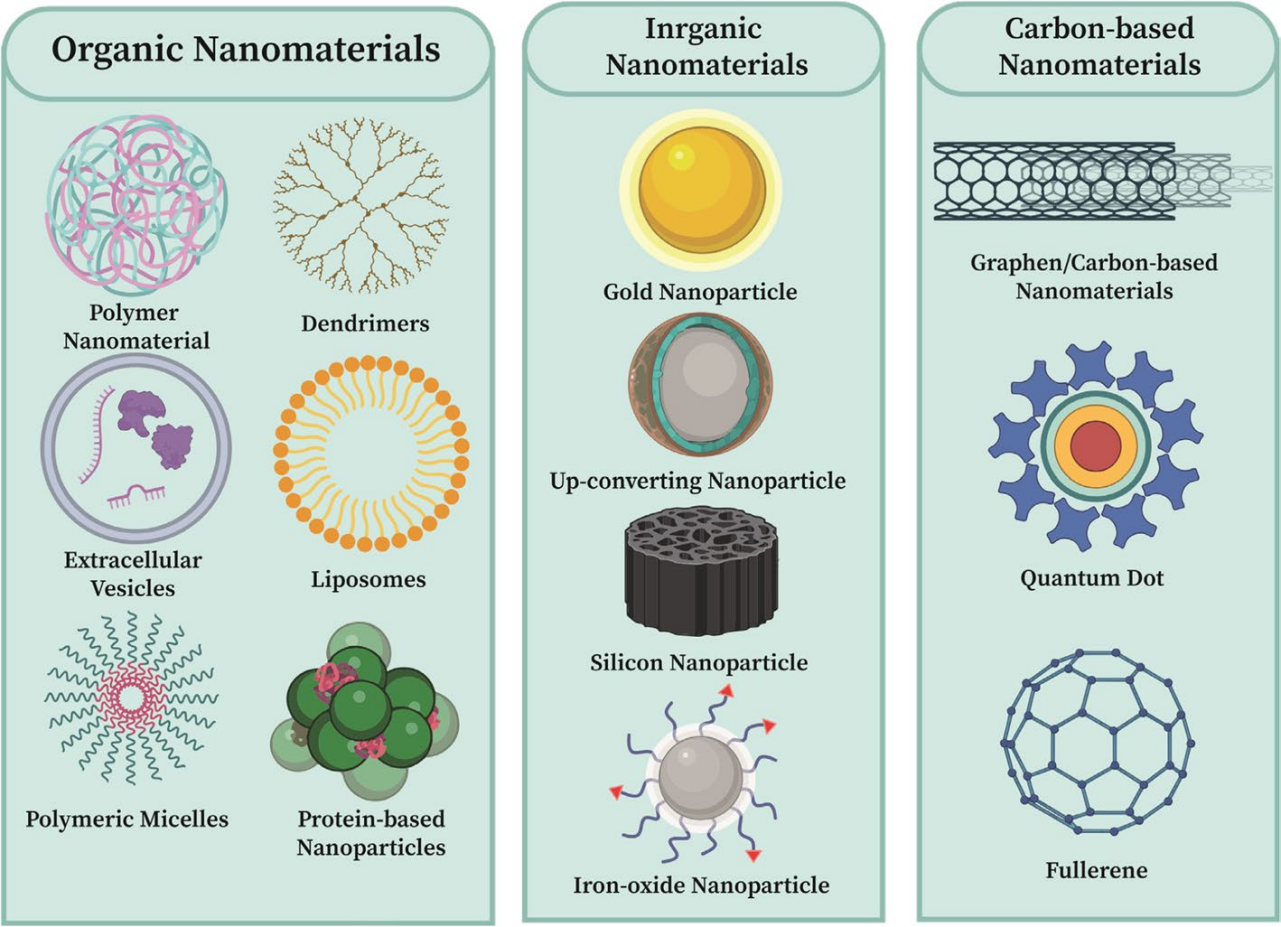
Classification of nanoparticles based on the chemical composition in three division. 1) Organic nanomaterials; 2) Inorganic nanomaterials; 3) Carbon-based nanomaterials

**Fig. 3:**
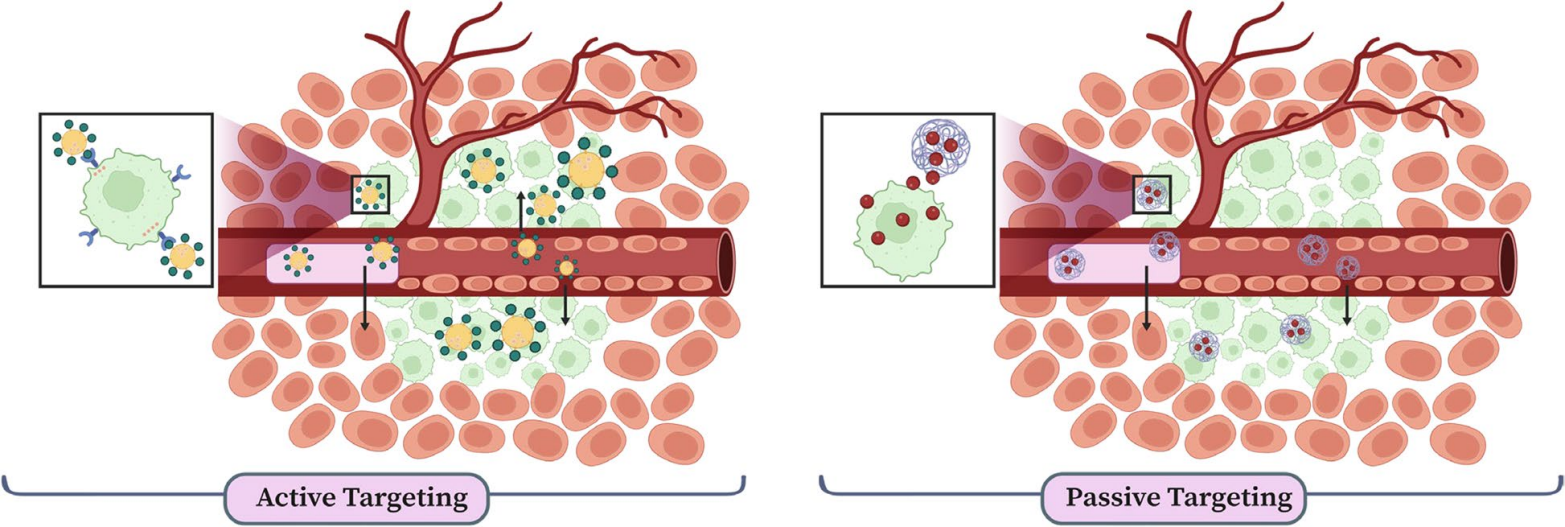
Illustration of passive and active tumor targeting used in the field of nanomedicine. Passive targeting is achieved by unique properties of tumor tissues, in which blood vessels are commonly leaky due to their unorganized structure and ineffective lymphatic drainage. The key factor driving this strategy is EPR effect. Hence, nanoparticles enter to the tumor tissue more easily than other healthy tissues. Active targeting involves surface functionalization of the nanoparticles with targeting moiety that have high affinity and specificity to recognize and bind to receptor and markers on the surface of cancer cells. In this approach, actively guiding the nanoparticles to the desired tissues improves the efficiency of drug delivery and therapeutic effect, as well as, minimizing side effects

Nanotechnology can generate new materials that act as valuable platforms for a wide range of applications, such as biosensing, bioimaging, nanomedicine, drug delivery, and nanotheranostics [17–22]. Nanoparticles propose great advantages to the field of multimodal imaging owing to their special features, including nanometer dimensions (1–100 nm), tunable imaging characteristics, and multifunctionality [23]. Based on the chemical compositions of nanoparticles, they are mainly classified into three categories: inorganic, organic, and carbon-based [24], as demonstrated in Fig. 2 Among all the properties of nanoparticles, size has a significant impact on tumor imaging. Nanoparticles show enhanced permeability and retention (EPR) effects in tumors due to their small size (see Fig. 3) [25]. These effects increase the local tumor concentrations of imaging agents. Moreover, blood circulation half-life, biodistribution, tumor targeting, and cellular uptake are remarkably associated with the size of nanoparticles [26]. One of the proposed procedures to gain quantitative information on the whole-body biodistribution is incorporating appropriate radionuclides in the nanoparticles [27, 28]. This approach is called "radiolabeling," and radioisotopes used for nuclear imaging and therapeutic purposes are listed in Table 2. The applications of radiolabeled nanoparticles as imaging probes have numerous benefits [28]. These nanoparticles amplify signals and improve contrast and sensitivity indices more than common radiotracers. Moreover, they can be easily conjugated with various biomolecules due to their large surface area for targeted cancer detection [29]. Furthermore, the novel concept of "nanotheranostics" emerged from the incorporation of both diagnostic and therapeutic moieties into one nanoplatform for improved targeted cancer management [30]. Generally, procedures used for radiolabeling nanoparticles are divided into four categories: a) chelator-based radiolabeling (indirect); b) direct bombardment of nanoparticles; c) chelator-free radiolabeling; d) mixture of nonradioactive and radioactive precursors used for the synthesis of nanoparticles [31]. Among these methods, chelator-free radiolabeling is a favorable choice because it can preserve the native physical features of nanoparticles, such as in vivo pharmacokinetics, surface charge, and particle size. Also, complicated conjugation chemistry is not required in chelator-free radiolabeling [32].

**Table 2:** Representative radionuclides used for radiolabeling of nanoparticles used in cancer imaging and therapy [33, 34]

Radionuclide	Half-life	Emission	Energy (KeV)	Application
**SPECT**				
^ 99m^Tc	6.02 h	γ	141	Imaging
^ 67^ Ga	3.26 d	γ	93, 185, 296	Imaging
^ 111^In	2.8 h	γ, Auger electrons	172,245	Imaging/Therapy
^ 131^I	8.02 d	γ, β^−^	364, 606	Imaging/Therapy
**PET**				
^ 18^F	109.8 min	β^+^	634	Imaging
^ 68^ Ga	67.7 min	β^+^	770, 1890	Imaging
^ 64^Cu	12.7 h	β^−^, β^+^	579, 653	Imaging/Therapy
^ 124^I	4.18 d	β^+^, γ	820, 1543, 2146	Imaging
^ 89^Zr	78.4 h	β^+^	396.9 (ave), 900 (max)	Imaging
**Other**				
^ 177^Lu	6.7 d	β^−^	140	Therapy
^ 188^Re	17.0 h	β^−^	795	Therapy
^ 90^Y	2.7 d	β^−^	935	Therapy
^ 32^P	14.3 d	β^−^	695	Therapy

Cancer therapy includes various treatment strategies such as surgery, chemotherapy, radiotherapy, and immunotherapy. Different factors (i.e., cancer type, grade, and stage) influence the therapeutic approaches, either alone or in combinations, used for cancer patients [35]. Engineered smart nanocarriers for tumor diagnosis and therapy, known as theranostic agents, find great potential, particularly in radiopharmaceutical therapy (RPT). Primarily, β-emitting and mostly potent α-emitting radionuclides are used in targeted delivery of radiation [36]. RPT is a new therapeutic technique offering several advantages over other approaches for the treatment of cancer. In comparison to radiotherapy, in targeted RPT, the radiation is delivered systematically inside the body, and cytotoxic radiation directly influences tumor cells and their microenvironment. Moreover, unlike other existing therapeutic approaches, targeting therapeutic agents is possible by using PET and SPECT imaging modalities for precise detection of RPT delivery. Minimal cytotoxicity and acceptable efficacy were observed for RPT [37]. Also, fast responses, a single or at most five injection doses, and less severe side effects are associated with the administration of RPT compared to the chemotherapy approach.

**Fig. 4:**
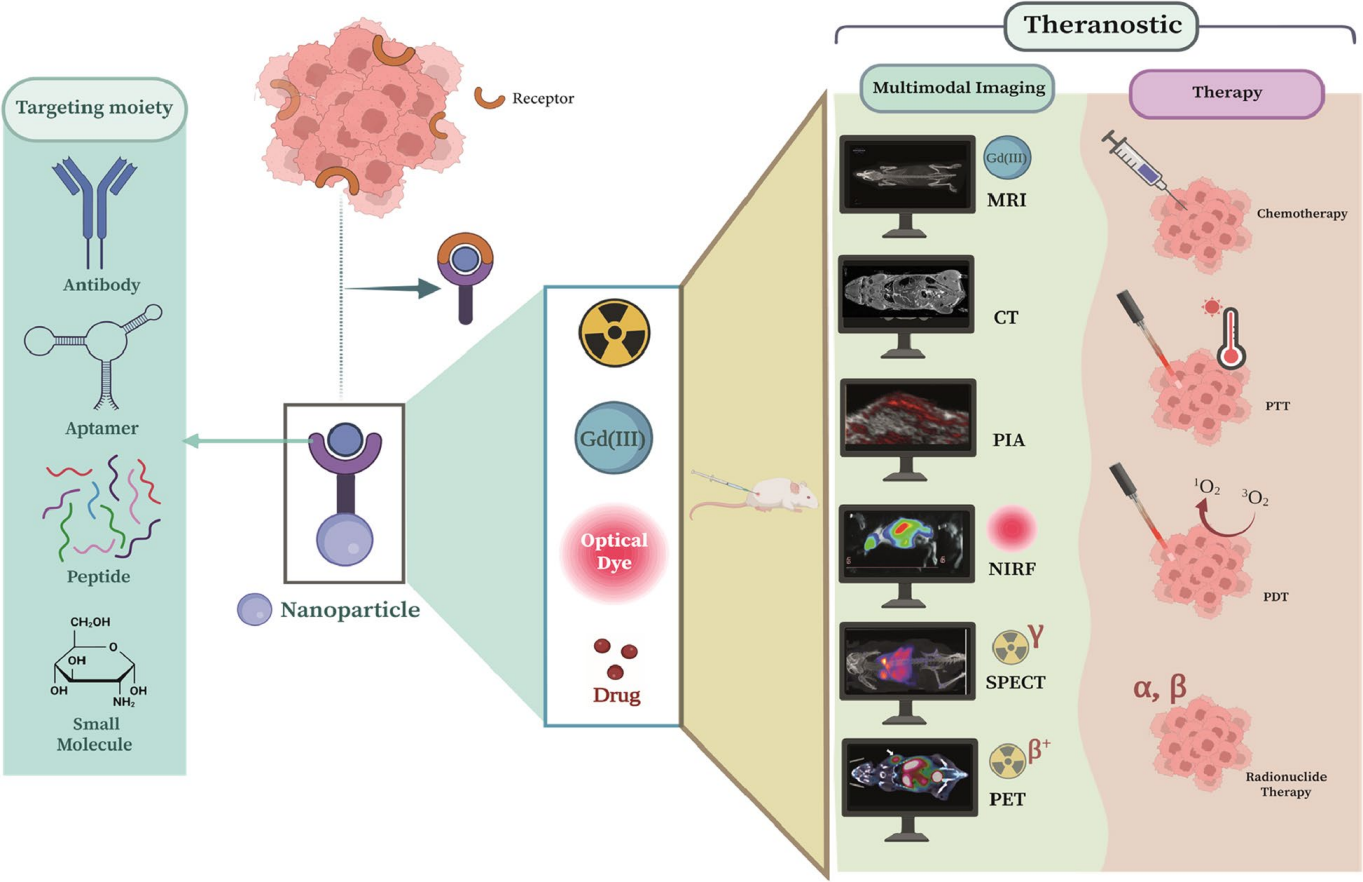
Development of multifunctional nanoparticles for targeted multimodal cancer imaging and theranostic application. Nanoparticles are functionalized with multiple contrast agents, therapeutic agent, targeting moiety (e.g., small molecules, aptamers, peptide, and antibodies), and then radiolabeled with radionuclide for multimodal cancer imaging and therapy

In this review, we present the latest progress in the design and synthesis of radiolabeled nanomaterials (with at least one dimension below 100 nm) for in vivo dual/multimodality cancer imaging and nanotheranostic applications (Fig. 4). Moreover, we discuss toxicity issues, challenges, and opportunities for future trends in developing desirable radiolabeled nanoparticles for multimodal imaging and nanotheranostic, as cutting-edge technologies, in preclinical and clinical purposes of cancer diagnosis and therapy.

### Radiolabeling of nanoparticles for multimodal imaging and theranostic applications

**Fig. 5:**
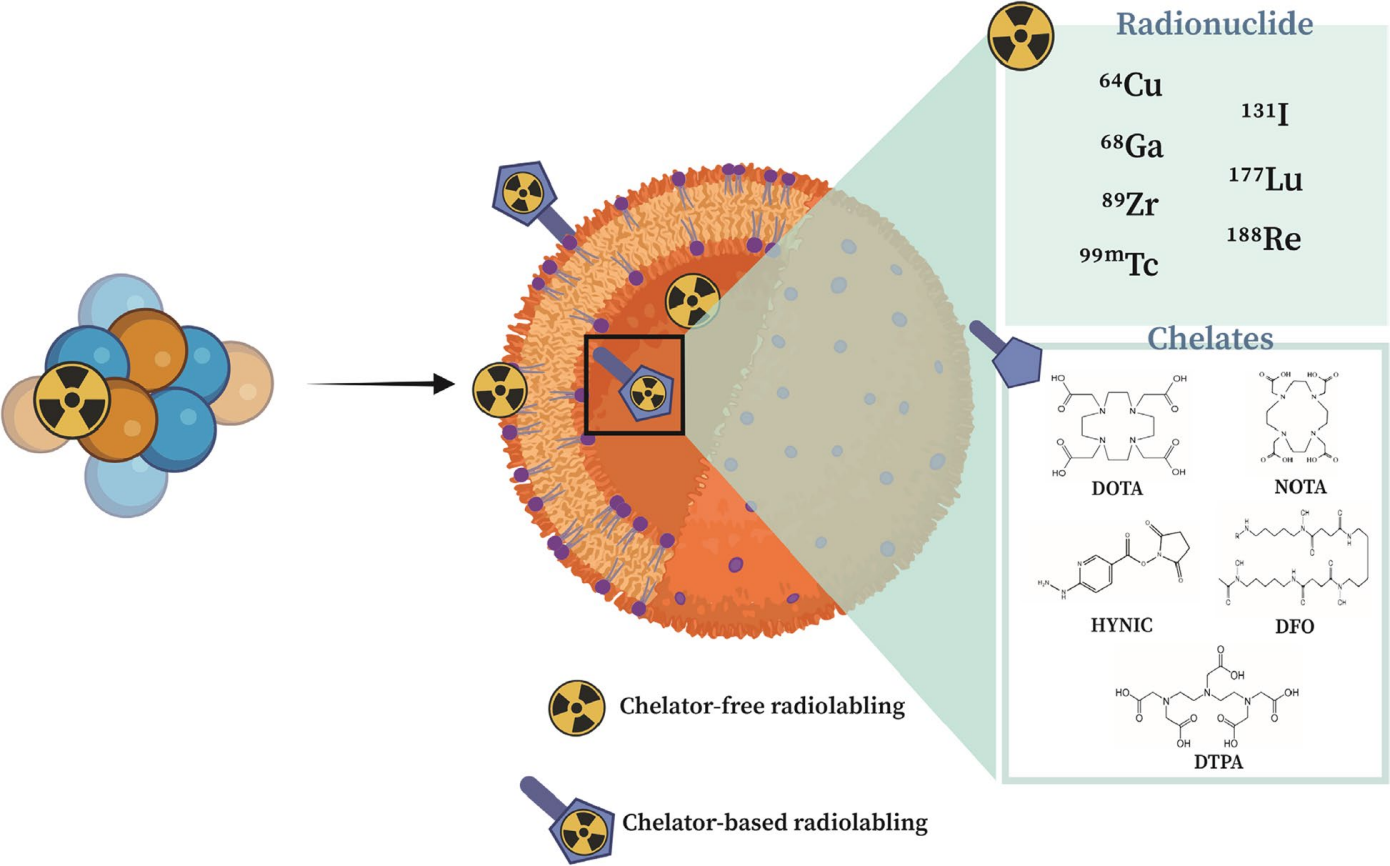
Radiolabeling of nanoparticles through two main strategies: chelator-based and chelator-free radiolabeling. Radionuclides and chelators typically used in radiolabeling of nanoparticles for multimodal cancer imaging and theranostic applications are depicted

Radiolabeling procedures are strategies used to attach radionuclides to nanoparticles. Many purposes can be achieved by this technique, such as understanding the biological behavior and pharmacokinetics of nanoparticles, non-invasive, real-time, and whole-body imaging, targeted therapy, and cancer image-guided therapy [38, 39]. The successful development of a radiolabeled nanoplatform basically relies on three divisions: 1) surface functionalization of the nanoparticle, 2) selection of an appropriate radioisotope, and 3) applying an efficient and reproducible radiolabeling procedure to combine Sections 1 and 2. An ideal radiolabeling method must be able to provide high radiochemical purity, good stability, and a simple, fast, low-cost, and robust strategy with minimal changes in the pharmacokinetic properties of nanoparticles. A radiation safety principle of "as low as reasonably achievable (ALARA)" represents guidelines for personal protection while working with radioactive materials [40], so it is preferred to do the radiolabeling process at the final step of construction. Here, we divide the radiolabeling strategies of nanoparticles for multimodality imaging and theranostic applications into two main categories, according to the use of chelators or not: 1) chelator-based radiolabeling 2) chelator-free radiolabeling (see Fig. 5).

Generally, most of the organic or inorganic nanoparticles were radiolabeled with chelator-based methods. Hence, the surface of the nanoparticles should first be covalently conjugated to an appropriate chelator [41, 42]. Different factors are associated with the selection of a particular chelator; the most important ones include the oxidation state and the physical properties of the radiometal ion [43]. The most common bifunctional chelators, which are linear or macrocyclic, are depicted in the Fig. 5. The formation of a stable coordination complex between nanoparticles and radiometal ion is required to avoid the detachment of radiometals from the nanoparticles, and it is possible via a strong binding of the radioisotope to the surface of the nanoparticles. Typically, this technique is applicable for nanoparticles, in which their surfaces are well-functionalized [44]. The considerable concerns in chelator-based radiolabeling are related to the multistep process needed for surface modification of nanoparticles with an appropriate bifunctional chelator, the increased time of production, as well as the alternation of physiochemical properties (i.e., the size, surface charge, and hydrophilicity of nanoparticles) after chemical modification with the chelator. Moreover, detachment of radiometal from the surface of nanoparticles might still be possible [31].

Chelator-free radiolabeling is a state-of-the-art approach that exploits the intrinsic physicochemical properties of nanoparticles. The revolution of this surface-based radiochemistry offers a great opportunity for the construction of radiopharmaceuticals for targeted multimodal imaging. In surface chemistry, the term "chemisorption" refers to the chemical binding of radioisotopes with functional groups on the surface of nanoparticles directly. Therefore, the chelator-free technique is a simple, fast, specific, and desirable alternative in which the physicochemical properties of nanoparticles are maintained without requiring a complicated surface-conjugated process with chelators [45]. Despite its advantages, chelator-free radiolabeling also presents certain limitations. Firstly, chelator-free methods avoid covalently linked chelators, but this means relying on the intrinsic properties of nanoparticles for radiolabeling. Surface modifications of nanoparticles significantly impact labeling yield. Some radioisotopes lack reliable molecular chelators, making it challenging to achieve stable labeling without them. The stability of chelator-free labeling varies across different radioisotope-nanoparticle combinations. Also, radiolabeling often requires harsh conditions (e.g., high temperatures, low or high pH) that may compromise nanoparticle stability. Maintaining stability during radiolabeling is crucial to prevent detachment of surface-bound labels. These issues can lead to inaccurate biodistribution imaging, affecting the reliability of results [42, 46].

### Radiolabeled nanoparticles for dual/multimodal *cancer* imaging

#### PET/CT and SPECT/CT

Positron emission tomography-computed tomography (PET/CT) is a reliable method used for the diagnostic imaging of multiple types of human cancers. In 1998, PET/CT imaging was first introduced and integrated anatomical data obtained by CT with functional data from PET, increasing sensitivity and allowing more efficient disease detection [47].

Most early studies have focused on the use of prostate-specific membrane antigen (PSMA) or gastrin-releasing peptide (GRP) receptors, as small-molecule based targeted probes, for prostate cancer [48, 49]. These agents indicated rapid body elimination through renal clearance and led to limited tumor penetration. Therefore, a polymeric nanoparticle has gained attention from Pressly et al. to prepare a PET/CT tracer for prostate cancer [50]. An amphiphilic comb-like nanoparticle was prepared and loaded with C-atrial natriuretic factor to target the natriuretic peptide clearance receptor. For radiolabeling of the complex with ^64^Cu, 1,4,7,10-tetraazacyclododecane-1,4,7,10-tetraacetic acid (DOTA) chelator was used. The results of PET/CT imaging and biodistribution of the targeted and nontargeted ^64^Cu-Comb nanoparticles on the CWR22 prostate cancer tumor model revealed low renal clearance, prolonged blood pool retention, and improved tumor penetration and tumor uptake. The tuned physiochemical properties and biological behavior of this targeted radiolabeled polymeric nanoparticle make it a promising candidate for prostate cancer PEC/CT scans.

Recently, a PET/CT probe based on amphiphilic polymer nanoparticles that were radiolabeled with ^68^Ga was synthesized for sentinel lymph node metastasis imaging [51]. The enhanced stability and radiolabeling yield of nanoparticles were associated with the increased rigidity of the used ligands. In this work, the importance of regulating the chelation efficiency and rigidity of the coordination structure of ^68^Ga-labeled nanoparticles in comparison with small-molecule probe-based ^68^Ga was investigated. The PET/CT scans demonstrated that the best differentiation of normal lymph nodes from tumor-metastasized sentinel lymph nodes was only feasible with the strongest rigidity of coordination structure. In 2019, Miedema et al. reported the application of PET/CT imaging based on radiolabeled nanoparticles in patients with advanced solid tumors for the first time [52]. They used CPC634, which is composed of docetaxel entrapped in a stabilized nanosized core-cross linked polymeric micelles by a covalent bond. CPC634 improved the EPR effect and tumor accumulation of the drug in comparison to typically administered docetaxel. Five patients with solid tumors received ^89^Zr-desferal-CPC634 and whole-body PET/CT scans were acquired at certain time intervals. Information gained from biodistribution studies indicated consistent and high retention of ^89^Zr-desferal-CPC634 in tumors and confirmed the EPR effect of CPC634 in humans, which is important to develop therapeutic agents for targeted tumor treatment. Another clinical study of radiolabeled nanoparticles for PET/CT scans in patients with esophageal cancer was reported in 2022 [53]. ^89^Zr-labeled high-density lipoprotein nanoparticle (HDL) was intravenously administered to nine patients with adenocarcinoma or squamous cell carcinoma. The findings proved safe administration of ^89^Zr-HDL and demonstrated accumulation of the radiotracer in tumors. HDL nanoparticles might have a potential opportunity for the delivery of anti-cancer drugs in the future. ^89^Zr-HDL nanoparticles were also investigated as a PET/CT tracer to monitor the response to immunotherapy in mice [54].

Lung ventilation-perfusion PET/CT offers useful information for the evaluation of regional lung function. This approach has revealed hopeful potential in different clinical strategies such as pulmonary embolism, radiotherapy, and pre-surgical assessment in patients with lung cancer [55]. In this regard, the chemical composition and physical properties of ^68^Ga-carbon nanoparticles as ventilation PET/CT probes were investigated and compared to Technegas®, a common clinical method used in the analysis of regional lung ventilation function [56]. Other nanoparticles (i.e., gold and platinum) were radiolabeled with 2-Deoxy-2-[^18^F] fluoro-D-glucose (^18^F‐FDG) and ^89^Zr for PET/CT imaging of tumor-bearing mice through active and passive targeting [57, 58].

SPECT/CT dual-modal imaging was introduced in 1977 to combine the advantages and overcome the limitations of each technique. This well-established procedure has been extensively used for clinical diagnosis applications. Because of the slow speed of developing SPECT/CT imaging probes, it is necessary to develop novel and efficient SPECT/CT contrast agents to improve the diagnosis of various diseases and clinical decision-making strategies [59]. For this purpose, a poly(lactic acid)-polyethylene glycol copolymer nanoparticle was conjugated to PSMA and radiolabeled with ^111^In through a chelator-based strategy (^111^In-DOTA-PEG-alkyne) [60]. The in vivo SPECT/CT scans of ^111^In-labeled targeted nanoparticles and ^111^In-labeled untargeted nanoparticle in tumor bearing mice revealed a modest positive impact on prostate cancer localization due to active targeting made by conjugating PSMA to nanoparticles than untargeted nanoparticles. Moreover, Li et al. reported the synthesis of a cost-effective nanoprobe for targeted tumor SPECT/CT scan [61]. They used low generation dendrimers, which their surface was covalently functionalized with PEGylated folic acid and DTPA chelator. Then the complex entrapped gold nanoparticles and radiolabeled with ^99m^Tc. This dual-contrast agent was tested for in vivo SPECT/CT imaging of cell-surface overexpressed folate receptor cancer in a mouse model.

#### PET/MRI and SPECT/MRI

Compared to PET/CT and SPECT/CT modalities, PET/MRI and SPECT/MRI, as the next generation of dual-modality imaging, have considerably improved the diagnostic process [62]. High spatial resolution, specificity, sensitivity, low dose of radiation, and soft-tissue penetration of MR imaging provide much more comprehensive information in comparison with CT [63]. Numerous magnetic materials, including Gd, Cu, Mn, Zr, and iron oxide, were used in the fabrication of PET/MRI probes [64–68]. Recently, Xie et al. developed a PET/MRI nanoprobe based on biocompatible melanin nanoparticles and ^124^I radionuclides [69]. WL12, a cyclic peptide with high affinity for programmed death protein ligand-1 (PD-L1), was selected for active targeting of cancer cells. PET/MRI imaging using this radiotracer in a mouse model with an A549 tumor demonstrated high tumor uptake and PD-L1-targeting, providing an excellent opportunity for PD-L1 therapy in patients with lung cancer.

Another PET/MRI and PET/CT modality of nanoprobes based on organic nanoparticles was constructed by Wen et al. [70]. Dopamine-melanin nanoparticles, which included biocompatible and biodegradable natural-produced dopamine, were used as a novel nanoplatform. The surface of nanoparticles was pegylated and loaded with a monoclonal antibody, trastuzumab. This antibody has a high affinity to target human epidermal growth factor 2 (HER2). The in vivo behavior and pharmacokinetics of this radiolabeled nanoprobe-(^124^I/^64^Cu, Mn)-Her-PEG-dMNPs were evaluated by PET/MRI and PET/CT imaging in patient-derived xenograft mouse models with gastric cancer. The valuable findings of this study indicated enhanced retention time of trastuzumab in tumors, low cardiac toxicity, and the possibility of following the therapeutic effect in real time via dual-modality imaging.

Radiolabeled superparamagnetic iron oxide nanoparticles (SIONPs) with various radionuclides have been greatly investigated for passive and active targeting with SPECT or PET/MRI imaging, particularly over the past decade [67, 71, 72]. Vascular endothelial growth factor A (VEGF-A) belongs to a glycoprotein family that can stimulate tumorigenesis when it is up-regulated. Bevacizumab, a recombinant humanized monoclonal antibody, inhibits angiogenesis by binding to VEGF-A and contributes to the treatment of various metastatic cancers [73]. In this respect, Tsoukalas et al. have developed a targeted radiotracer, ^99m^Tc-labeled functionalized IONPs conjugated to bevacizumab, for SPECT/MRI imaging evaluation of VEGF-overexpressing in M165 tumor-bearing mice [74]. They proposed therapeutic radionuclide Rhenium-188, which shows similar chemical properties to ^99m^Tc, for assessment of nanotheranostic potential of functionalized IONPs conjugated to bevacizumab.

#### PET/OI and SPECT/OI

In spite of the many advantages of OI, it suffers from low tissue penetration, which leads to poor quantification and limited clinical utility. Therefore, the complementary nature of radionuclide-based imaging modalities (SPECT or PET) and OI reinforces the efficacy of cancer diagnosis.

Human chimeric antigen receptor (CAR) T cell labeling allows dynamic monitoring of cell tracking, which is an enticing prospect in cancer therapy. Recently, Harmsen et al. reported a clinically translatable dual-modal PET/NIRF nanotag with outstanding results such as high radiolabeling and loading yield of nanoparticles, stable intracellular trapping, efficient delivery to cancer cells, and releasing [75]. Biocompatible fluorescent silica nanoparticles were radiolabeled with ^89^Zr and then labeled CAR T cells via heparin-protamine. All these major materials, including ^89^Zr, fluorescent silica nanoparticles, protamine, and heparin, have been proven for clinical use. This nanotag enabled CAR T cell tracking by PET/NIRF imaging up to one-week post-adoptive cell transfer in an ovarian peritoneal carcinomatosis model.

Tumor cell-derived exosomes (TEx) have emerged as a natural drug delivery nanoplatform due to their endogenous origin, nontoxicity, and biocompatibility. Their clinical translation for multimodal application is related to their surface functionalization; however, this process is still challenging due to their small size and complicated surface chemistry. For the first time, an exosome-based multimodal imaging agent (SPECT/NIRF) for colon cancer has been engineered by Jing et al. [76]. They used a simple, rapid, low-cast, and high-yield hydrophobic insertion approach to label TEx with fluorescent dye (Cy7) and ^99m^Tc radionuclide without changing the morphology and natural properties of exosomes.

Quantum dots (QDs) are nanoscale inorganic fluorescent crystals with unique optical properties, including high quantum yield, high brightness, high stability, low photo-bleaching, size-tunable absorption, and emission spectra [77, 78]. PET/NIRF nanoprobe was prepared by radiolabeling QDs with ^64^Cu‌ through a chelator-based strategy and using RGD peptides as the targeting agent [79]. Moreover, optical nanocrystals (i.e., QDs, upconversion nanoparticles) have been encapsulated into mesoporous silica nanoparticles for imaging applications [80, 81]. In Chen et al.'s research, a novel PET/NIRF modality-based mesoporous silica nanoparticle was synthesized by conjugating a NIRF dye and a human/murine chimeric IgG1 monoclonal antibody on the surface of nanoparticles and radiolabeled with ^64^Cu [82]. Efficiently and specifically, tumor targeting and the pharmacokinetics of this dual-modal imaging agent were evaluated in 4T1 breast tumor-bearing mice.

Raman imaging is derived from the phenomenon of Raman scattering, which involves the inelastic scattering of photons by molecules that have been stimulated to higher energy states. Nevertheless, the Raman scattering signal poses a significant challenge due to its inherent weakness and the arduous task of its acquisition. This is primarily due to the scarcity of photons that undergo inelastic scattering, which accounts for a minuscule fraction of around one tenth of the total scattered photons. The initial investigation of surface-enhanced Raman scattering (SERS) was conducted by Fleischmann et al. [83]. Their study revealed that the Raman signal of pyridine molecules, when deposited on the surface of a nanoscale, rough silver electrode, exhibited a relatively high intensity. Additionally, they observed that alterations in the applied potential on the electrode resulted in corresponding changes in the intensity of the Raman signal. Following this, Jeanmaire et al. made the observation that the Raman signal experienced a substantial enhancement of around six orders of magnitude as a result of the surface roughness shown by nano-scale gold, copper, silver, and other similar materials [84]. Due to the distinctive characteristic of SERS, the utilization of SERS for imaging purposes is unaffected by autofluorescence, a prevalent obstacle encountered by alternative OI techniques [85]. Nevertheless, like other OI methodologies, the use of SERS is also impeded by its constrained ability to penetrate deeply into the sample. In order to address this issue, Wall et al. employed a discreet approach to develop a SERS nanoparticle tagged with ^68^ Ga for the purpose of conducting simultaneous PET and SERS imaging of tumors [46]. The PET-SERS nanoparticles were employed in several investigations, such as lymph node tracking, surgical guidance for lymph node excision, and cancer imaging subsequent to intravenous administration, serving as a proof-of-concept. The considerable concentration of PET-SERS nanoparticles in the reticuloendothelial system (RES) resulted in significant uptake in healthy RES tissues and comparatively decreased uptake in malignant tissues during both SERS mapping and PET imaging of liver tumors.

#### PET/PAI

Photoacoustic imaging (PAI) is a recently developed non-invasive biomedical imaging technique that relies on the photoacoustic phenomenon. When biological tissues are exposed to pulsed laser radiation, a portion of the absorbed energy is transformed into heat, resulting in periodic thermal contraction and expansion of the lighted tissues. This phenomenon leads to the generation of ultrasonic waves. PAI combines the advantageous contrast effectiveness of OI with the deeper penetrability of ultrasonic imaging. Consequently, PAI has the capability to offer high-contrast imaging of the intricate structure of tumors located deep inside the body. Additionally, PAI can provide valuable anatomical, functional, and metabolic details for the purpose of cancer detection [86, 87].

A variety of PAI contrast agents have been formulated and fabricated, including inorganic nanomaterials that include noble metals, boron nitride, and carbon-based nanomaterials, as well as organic nanomaterials comprising NIR-responsive tiny molecules as well as semiconducting polymeric nanostructures [88, 89]. In order to combine the excellent contrast and good spatial resolution of PAI with the quantitative capabilities of nuclear medicine imaging (NMI), some nanomaterials have been effectively tagged with radionuclides. This enables the utilization of dual-modality imaging, combining NMI and PAI, which has demonstrated exceptional diagnostic outcomes in both laboratory settings (in vitro) and living organisms (in vivo) [90–93]. The Cheng research group has produced innovative nanoprobes, namely Au tripods with a size smaller than 20 nm. These nanoprobes exhibit meticulously regulated shapes and possess distinct visible and near-infrared absorption properties [12]. The Au-tripods that were PEGylated underwent further conjugation with cyclic c(RGDfC) peptide, resulting in RGD-Au-tripods. These RGD-Au-tripods were then radiolabeled with radionuclide ^64^Cu. The purpose of this was to assess the effectiveness of tumor targeting and imaging in a subcutaneous α_v_β_3_-positive U87MG glioblastoma xenograft model. Both PAI and PET demonstrated significant tumor accumulation and a pronounced contrast between the tumor and adjacent muscle tissues. In particular, the radioactivity accumulation in the tumor reached 7.9% ID/g after 24 h after injection, which was shown to be more than three times more than the accumulation seen in the blocking group (2.6% ID/g). The acquisition of functional and molecular data from the tumor was accomplished using PAI, which exhibited a strong correlation with the quantification achieved by PET and effectively addressed the limitations of PET's spatial resolution.

In a recent study, the Chen group documented the effective manufacturing of CuS-ferritin nanocages (CuS-Fn NCs) that included ultrasmall CuS nanoparticles within the cavity of Fn NCs [33]. These nanocages exhibited favorable biocompatibility, robust near-infrared absorbance, and pronounced photoacoustic contrast. The researchers utilized uniform CuS-Fn NCs that were radiolabeled with ^64^Cu as nanoprobes featuring dual-modality capabilities for PET/PA imaging. These nanoprobes were employed to visualize human glioblastoma U87MG-bearing nude mice, demonstrating excellent sensitivity and precise spatial resolution. Following intravenous administration, there was a consistent rise in the PA signal inside the tumor area, indicating a continual accumulation of CuS-Fn NCs. This observation was further validated by the utilization of 3D-PAI techniques. The results obtained from the quantitative analysis using PET were in agreement with the findings from the pharmacokinetic study, indicating that around 10% of the injected dose per gram (ID/g) of ^64^CuS-Fn NCs accumulated in the U87MG tumors after 8 h of administration. Subsequently, there was a little reduction in tumor uptake within 24 h. Furthermore, the use of CuS-Fn NCs in photothermal therapy has been facilitated by their notable photothermal conversion efficiency. The efficacy of these NCs in cancer treatment has been substantiated by their exceptional cancer therapeutic efficiency as well as their favorable biocompatibility, as demonstrated in both in vitro and in vivo experiments.

#### PET or SPECT /Cerenkov luminescence imaging

Cherenkov radiation refers to the phenomenon of electromagnetic radiation being produced when a charged particle traverses a dielectric medium at a velocity exceeding the phase velocity of light in that particular medium. For instance, some positron emitters such as ^13^N and ^18^F, as well as electron emitters like ^90^Y and ^32^P [34], exhibit detectable Cherenkov emission. Additionally, isotopes ^131^I and ^18^F have been successfully seen in human subjects to demonstrate their diagnostic utility [94, 95]. Nevertheless, the utilization of Cherenkov luminescence (CL) imaging is constrained by its restricted penetration depth, mostly attributed to its short wavelength. This limitation has prompted researchers to explore the integration of CL with NMI. It is noteworthy that nanomaterials such as carbon dots, upconversion nanoparticles, Au nanoparticles, and quantum dots have the ability to absorb Cherenkov light of short wavelengths released by radionuclides, then emit near-infrared light with longer wavelengths [96, 97]. The physical phenomenon under consideration is referred to as Cerenkov resonance energy transfer (CRET), which has garnered significant interest owing to its ability to penetrate deeper, exhibit a lesser background signal, and achieve higher levels of detection sensitivity [96]. The Chen research group successfully manufactured radioactive [^64^Cu]CuInS/ZnS QDs by integrating ^64^Cu into the CuInS/ZnS nanostructure. This development enables the utilization of CRET and PET techniques for luminescence imaging of U87MG glioblastoma xenograft tumors [98]. The findings indicated that the nanoprobe exhibited favorable luminescence imaging capabilities for malignancies without the need for external light stimulation. The results of the quantitative evaluation of PET images revealed that the uptake of tumors exhibited a notable rise, reaching a maximum value of 10.8% injected dose per gram (ID/g) at 18 h after injection.

Nanomaterials, including noble metals, including silver, gold, and platinum, have the capacity to absorb Cherenkov light because of the localized surface plasmon resonance (LSPR) effects. Consequently, these materials have promise for luminescence imaging by CRET. An instance of utilizing ^64^Cu as both the PET tracer and the energy donor to stimulate gold nanoclusters for generating NIR fluorescence signals has resulted in the development of ^64^Cu-doped gold nanoclusters. These nanoclusters have been employed for CRET and PET-based near-NIR imaging of U87MG tumor-bearing mice. Notably, the accumulation of radioactivity in the tumor reached a substantial level of 14.9% injected dose per gram (ID/g) after 40 h [97].

#### Multimodal imaging

In recent years, extensive efforts in preclinical biomedical research have been devoted to engineer novel multimodal imaging systems using functional nanoparticles to improve the accuracy and sensitivity of cancer detection at a very early stage. To design this multimodal imaging agent, upconversion nanoparticles (UCNPs), especially lanthanide-doped nanocrystals, have drawn great attention as a promising next generation of fluorescent nanoprobes. In these nanoparticles, near-infrared (NIR) radiation is converted to visible light by a mechanism called "upconversion luminescence". Therefore, UCNPs indicate exceptional chemical and optical properties, including the absence of autofluorescence and photoblinking, high photostability, narrow emission peaks, and low toxicity in comparison to common down-conversion fluorescent agents (i.e., fluorescent dyes, QDs) [99–102]. A biodegradable and potent pentamodal imaging nanoprobe based on fluoromagnetic UCNPs (NaGdF4:Yb,Er) was prepared, in which the apo-human serum transferrin protein was conjugated on the surface of nanoparticles to actively target the transferrin receptors overexpressed on various cancer cells [103]. The nanosystem was radiolabeled with ^99m^Tc and demonstrated high radiochemical purity (∼95%) and excellent in vitro stability. For activation fluorescence and photothermal imaging, deep tissue penetration at the 980 nm wavelength was used to excite UCNPs. The Yb and Gd elements in the composition of nanoprobe have led to CT and T_1_-weighted MR imaging. Tumor accumulation, active targeting, renal clearance, and near-ideal in vivo behavior were confirmed using MRI/thermal-camera/SPECT/CT imaging in 4T1 tumor-bearing mice. Furthermore, UCNPs were coated with red blood cell membrane for upconversion fluorescence imaging (UCL)/MRI/PET imaging of triple-negative breast cancer-bearing mice [104]. 1,2-distearoyl-sn-glycero-3-phosphoethanolamine-N-[folate(polyethylene glycol)-2000] (DSPE-PEG-FA) was conjugated to cell membranes to enhance tumor targeting.

Since IONPs could serve as contrast agents in MRI, they are an attractive nanoplatform for the fabrication of multimodal imaging nanoprobes. In this respect, PET/NIRF/MRI, magnetomotive ultrasound, and PET/CT/MR imaging based on IONPs were developed, in which ^64^Cu and ^68^ Ga were used for radiolabeling, respectively [105, 106]. In the first study, IONPs were coated with human serum and labeled with Cy5.5 fluorescent dye. Biodistribution of the prepared trimodal imaging agent was assessed in a subcutaneous U87MG xenograft mouse model. The results indicated a prolonged circulation half-life, high accumulation in lesions, and low uptake of the radiotracer by macrophages at the tumor sites [105].

**Fig. 6:**
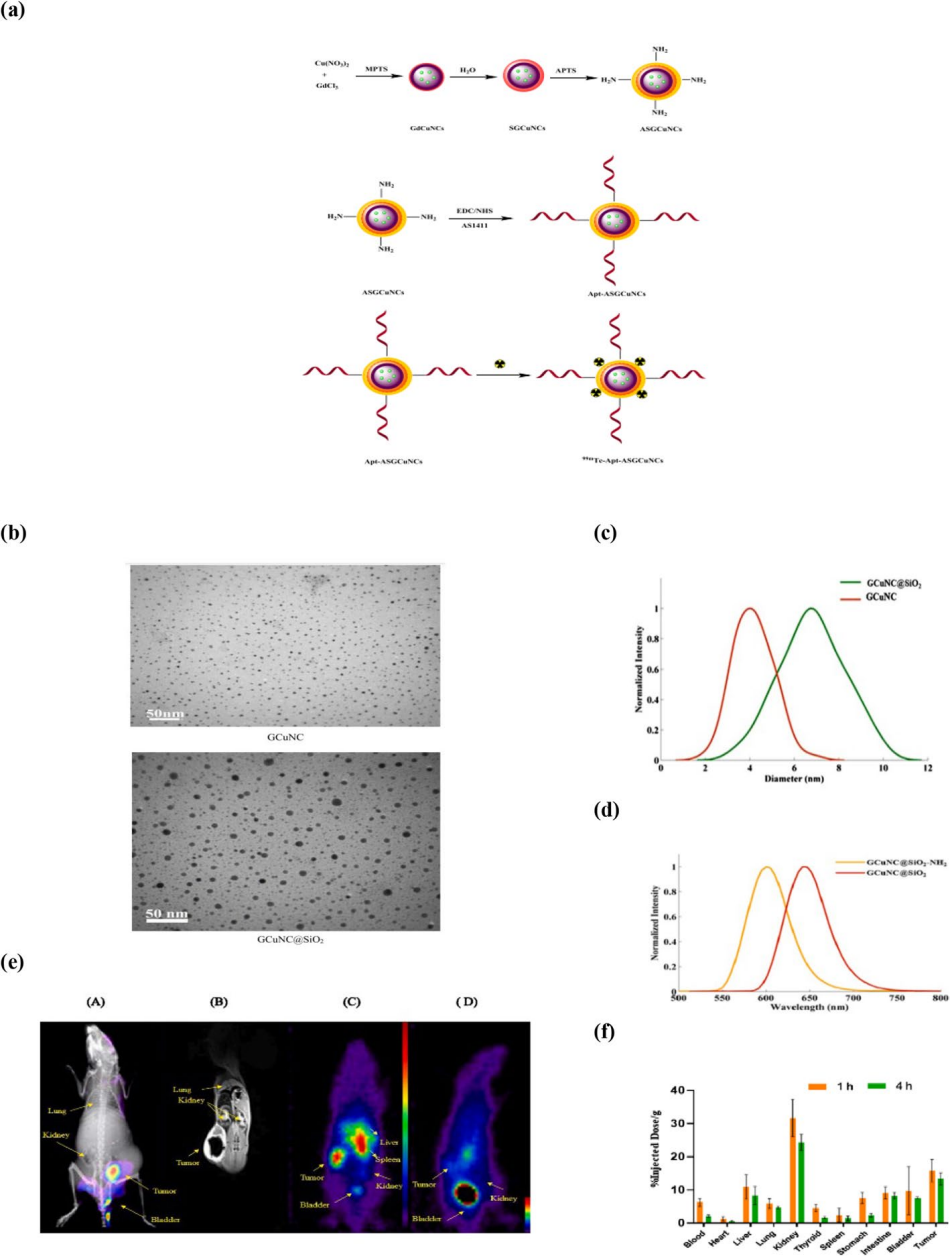
**a** Schematic representation of a procedure for synthesizing an innovative multimodal imaging agent. This process includes the synthesis of Gadolinium-Copper nanoclusters (GCuNCs), surface modification, AS1411 aptamer conjugation, and ^99m^Tc radiolabeling. **b** and (**c**) are transmission electron microscopy (TEM) visuals and analysis of particle size of GCuNC and GCuNC@SiO_2_, respectively. **d** The Photoluminescence (PL) spectral analysis of both GCuNC@SiO_2_ and its aminated variant, GCuNC@SiO2-NH_2_. **e** Comprehensive imaging investigations on BALB/c mice with 4T1 tumors (located on the right flank) one hour post intravenous administration of Apt-ASGCuNCs. The figure illustrates: **A** The results from fluorescence imaging, **B** Images obtained from magnetic resonance imaging, **C** SPECT/CT images captured one hour post injection of ^99m^Tc-Apt-ASGCuNCs without and (**D**) with the simultaneous administration of AS1411 aptamer (aptamer blocking). **f** The biodistribution pattern of ^99m^Tc-Apt-ASGCuNCs across various tissues at two distinct time periods (measured in dose per gram of tissue and *P-value* < 0.05).  (Reproduced with permission from ref. [107])

Recently, our group reported a novel multimodal imaging agent based on copper nanoclusters (CuNCs) [107]. Because of the intrinsic optical properties of nanoclusters, CuNCs were used as fluorescent agents and nanoplatforms to gather other contrast agents. Amino-modified silica-coated gadolinium-CuNCs were synthesized and conjugated to the AS1411 aptamer, then radiolabeled with ^99m^Tc for fluorescence imaging, MRI, and SPECT/CT of 4T1 tumor-bearing BALB/c mice. The in vitro and in vivo studies revealed positive results about the prepared radiotracer, which made it interesting for future multimodality imaging applications (see Fig. 6).

A novel and very versatile nanoporphyrin platform has been successfully engineered, demonstrating exceptional capabilities in the simultaneous chelation of ^64^Cu^2+^ and Gd^3+^. This platform has great promise as a multi-modal imaging agent, offering combined PET/NIRF/MRI functionality [108]. The disulfide-crosslinked nanoparticles (CNPs) synthesized in this investigation were in an inactive condition while circulating in the bloodstream, hence reducing the level of fluorescence signal originating from the background. Following accumulation at the tumor site through the EPR effect, the CNPs faced dissociation as a result of disulfide bond breaking mediated by glutathione (GSH). Consequently, the fluorescent signal was triggered. As a result, the mean fluorescence signal of CNPs at the tumor location exhibited a 15.2-fold increase compared to the signal observed in muscle tissue and a 3.1-fold increase compared to non-crosslinked nanoparticles (non-CNPs) at the tumor site 24 h after injection. In a similar vein, the dissolution of gadolinium (III)-chelated CNPs at the tumor location facilitated the interaction between gadolinium ions and neighboring protons, leading to a notable enhancement in MRI contrast. In the field of PET imaging, it was shown that the accumulation of ^64^Cu-labeled nanoparticles in tumors reached its peak level 16 h after injection. Furthermore, the PET signal mostly originated from the tumor location, exhibiting an extremely low background signal after 24 h. The findings from these experiments collectively indicate the significant capabilities of nanoporphyrin as tri-modal imaging agents for tumor diagnosis, specifically in the context of PET/NIRFI/MRI. A novel nanoplatform has been created, utilizing ultra-small (< 10 nm) water-soluble melanin nanoparticles (MNPs), which possess a diverse array of clinically significant functionalities. This nanoplatform exhibits photoacoustic properties suitable for PAI as well as a high affinity towards specific metallic ions (Fe^3+^ for MRI and ^64^Cu^2+^ for PET) [109]. The MNPs were conjugated with a cyclic c(RGDfC) peptide, resulting in their ability to specifically target tumor cells that overexpress the α_V_β_3_ integrin. This conjugation also led to a significant concentration of the MNPs in U87MG tumors. By integrating the physiological data obtained through PET with the whole-body imaging capacity, the functional and molecular information derived from PAI with its high spatial resolution, and the anatomical data provided by MRI, the PET/PAI/MRI technique allows for the non-invasive acquisition of molecular as well as anatomical details at various depths.

Bi_2_Se_3_ nanosheets decorated with FeSe_2_ nanoparticles (FeSe_2_/Bi_2_Se_3_) were utilized as a representative instance of tetra-modal imaging probes. These probes were radiolabeled with ^64^Cu to enable PET/MR/CT/PA multimodal imaging of tumor-bearing mice. The FeSe_2_ component of the probes demonstrated high *r2* relaxivity, while the Bi_2_Se_3_ component exhibited X-ray attenuation ability. Additionally, the FeSe_2_/Bi_2_Se_3_ probes displayed strong NIR optical absorbance [110]. Following a 24-h post-injection period, notable changes were seen in several imaging modalities at the tumor locations. These changes included a surprising darkening effect in MRI, a considerable rise in Hounsfield unit values in CT, much stronger photoacoustic signals, and evident tumor contrast in PET. The consistent imaging results obtained in this study demonstrate the significant tumor absorption of FeSe_2_/Bi_2_Se_3_-PEG through the EPR effect. These findings underscore the potential applicability of two-dimensional composite nanostructures in the field of cancer imaging. In a recent study, the Cai group successfully created a tetramodal imaging nanoprobe for PET/fluorescence/CL/CRET imaging. This was achieved by assembling CuS nanoparticles onto the surface of hollow mesoporous silica nanoshells packed with porphyrin molecules labeled with ^89^Zr. The resulting nanoprobe exhibited the combined benefits of various imaging modalities, enabling the swift and precise identification of tumors [111].

#### Radiolabeled nanoparticles for theranostic applications

The application of nanoradiopharmaceuticals as biologically active nanoparticles labeled with diagnostic or therapeutic radionuclides opens a new horizon in the fields of radiopharmacy, nuclear medicine, and oncology [112]. In the modern healthcare management system, the application of radiopharmaceuticals has increased in different types of cancers and diseases and gained more and more attention from researchers. In 2002, the term theranostics’ as a single platform coupling both therapy and diagnostic imaging properties was introduced by Funkhouser et al. [113]. Some of the advantages of the theranostics system are better pharmacokinetics, higher cellular uptake, lower cytotoxicity, and simultaneous and targeted diagnosis and therapy. Therefore, the prediction of therapeutic response for a particular disease could be more accurate using the theranostic approach. Moreover, the term ‘personalized medicine’ was presented in medicine for the new paradigm of theranostics and summarized by the statement, "If you can see it, you can treat it" [114]. For the first time, ^131^I was used clinically for thyroid cancer and thyroid-related diseases like hyperthyroidism, and Grave’s in a theranostics approach almost 90 years ago.

Generally, radionuclides emit α, β^−^, β^+^ particles or γ-rays. β^+^ and γ-ray emitter radiotracers are responsible for diagnosis imaging, whereas α and β^−^ emitters used for treatment of various diseases and malignancies. For SPECT imaging, ^99m^Tc, ^67^ Ga, ^111^In, ^123^I and ^131^I are commonly used, whereas ^18^F, ^64^Cu,^68^ Ga, ^89^Zr and ^124^I are routinely applied for PET imaging. For therapeutic applications, two categories of radionuclides, β^−^ emitting (Lutetium, Iodine, and Yttrium) and α-emitting ones (Radium, Actinium, Bismuth, and Astatine), are usually used [115, 116].

Individual radionuclides can be used for imaging and/or therapy based on the type of their emission. Moreover, certain combinations are reported and listed in Table 3 as theranostic pairs for both imaging and therapy. Numerous theranostic pairs have been developed for the diagnosis and treatment of various cancers, such as prostate cancer, neuroendocrine tumors, bone metastases, and glioblastoma [117–121]. Since radionuclides like ^177^Lu and ^131^I emit both β^−^ and γ, they could be used as diagnostic and therapeutic agents simultaneously. Moreover, nuclear physicians and the pharmaceutical industry are increasingly intrigued by α-emitters, which hold promise for theranostic applications. Alpha emitters such as ^211^At, ^213^Bi/^212^Bi, ^223^Ra, ^225^Ac, and ^227^Th are currently under investigation in clinical settings. An intriguing isotope is ^212^Pb, which generates the alpha-emitting isotope ^212^Bi in vivo. For theranostic applications involving α-emitters, potential pairings include ^68^ Ga with ^225^Ac or ^203^Pb with ^212^Pb [36, 122, 123]. Furthermore, the nature of nanoparticles offers exceptional opportunities to bring drugs and theranostic radionuclide pairs together for synergistic therapeutic effects.

**Table 3:** Examples of radiotheranostic pairs routinely used in nuclear medicine or under clinical studies

Diagnostic radionuclide	Radiopharmaceutical	Therapeutic radionuclide	Radiopharmaceutical	Reference
^99m^Tc	DMSA^a^and MAG3^b^	^188^Re	DMSA^a^ and MAG3^b^	[124, 125]
^68^Ga	PSMA^c^-11	^177^Lu	PSMA^c^-617	[126, 127]
^68^Ga	NeoBOMB1^d^	^177^Lu	NeoBOMB1^d^	[128]
^68^Ga, ^111^In	DOTATATE, DOTATOC	^90^Y/^177^Lu	DOTATATE, DOTATOC	[129, 130]
^64^Cu	Bombesin analog, PSMA^c^	^67^Cu	Bombesin analog, PSMA^c^	[131, 132]
^123^I	MIBG^e^	^131^I	MIBG^e^	[133]
^68^Ga	DOTATATE, DOTATOC	^225^Ac	DOTATATE, DOTATOC	[134]
^68^Ga	PSMA^c^-617	^225^Ac, ^213^Bi	PSMA^c^ -617	[135]
^68^Ga	Pentixafor	^177^Lu	Pentixafor	[135]
^203^Pb	VMT-α-NET	^212^Pb	VMT-α-NET	[136]

^a^DMSA: Dimercaptosuccinic acid

^b^MAG3: Mercaptoacetyltriglycine

^c^Prostate-specific membrane antigen

^d^NeoBOMB1: Gastrin-releasing peptide receptor antagonist

^e^Metaiodobenzylguanidine

Iodine-131 (^131^I) is the most routinely used iodine radioisotope for radionuclide therapy and imaging of thyroid cancer and diseases due to its high affinity for collection in the thyroid gland. This radioisotope decays γ-rays and β^−^ particles, which are responsible for SPECT imaging and therapeutic effects, respectively [137]. Theragnostic molecular probes labeled with ^131^I have been developed for different types of cancer treatment [138]. In a research, scientists prepared ^131^I-labeled polyethylenimine-entrapped gold nanoparticles for SPECT/CT imaging and radionuclide therapy [139]. The PEGylated polyethylenimine is conjugated to a tumor-specific ligand, which has high affinity and specifically binds to MMP2. 3-(4-hydroxyphenyl) propionic acid-OSu (HPAO) was used for ^131^I radiolabeling. The multifunctional nanoparticles were evaluated for targeted imaging and therapeutic effects on various MMP2-overexpressed tumors and indicated excellent theranostic potential. In 2022, researchers synthesized ^131^I-labeled bovine serum albumin-modified copper sulfide nanoparticles for SPECT/CT imaging, radiotherapy, and photothermal therapy (PTT) of anaplastic thyroid carcinoma [140]. In vitro and in vivo assessments demonstrated the low toxicity and good biocompatibility of this theranostic nanoprobe. Particularly, the results confirmed the potential of combined PTT and radiotherapy in inhibiting tumor growth compared to monotherapy. In a more recent study, a multimodal breast cancer theranostics nanosystem was reported for SPECT/NIRF dual-modal imaging, radiotherapy, PTT, and photodynamic therapy (PDT) [141]. For this purpose, a new category of organic optical nanomaterials, known as semiconducting polymer nanoparticles with unique optical properties and good biocompatibility, were used and radiolabeled with ^131^I. High diagnostic efficiency and therapeutic effects in inhibiting tumor growth, liver, and lung metastasis were observed.

Many other developed nanotheranostics platforms, in which ^177^Lu, ^64^Cu, ^99m^Tc, ^125^I were used for radiolabeling, are listed in Table 4.

**Table 4:** Recent radiolabeled nanoprobes for targeted cancer multimodal imaging and theranostic applications

Class	NPs type	Targeting moiety	Radionuclide /Chelator	Multimodal Imaging	Therapy	Theranostic	Reference
**Organic NPs**							
	Melanin NPs	Melanin	^64^Cu/ chelator-free	PET/CT	Radionuclide therapy	Yes	[96]
	Semiconducting polymer NPs	-	^131^I/ chelator-free	NIRF/SPECT	Radionuclide therapy PTT PDP	Yes	[93]
	Tumor cell-derived exosomes	Inherent targeting	^99m^Tc/ chelator-free	NIRF/SPECT	-	No	[67]
	Peptide NPs	RGD-mitochondria targeting peptide (KLAK)	^99m^Tc /DTPA	NIRF/SPECT/CT	Therapeutic potential	Yes	[94]
	Metal − phenol Nps	Folic acid	^111^In/ chelator-free ^64^Cu/ chelator-free	SPECT/CT PET/CT	-	No	[95]
	Hyaluronan-cholesteryl hemisuccinate NPs	curcumin	^99m^Tc/ TPGS (D-a-tocopheryl polyethylene glycol succinate)	SPECT/CT	Chemotherapy	Yes	[97]
	PEI-entrapped AuNps	Buthus martensii Karsch chlorotoxin	^131^I /HPAO	SPECT/CT	Radionuclide therapy	Yes	[91]
**Inorganic NPs**							
	IONPs	PSMA	^68^Ga/DOTA	PET/MRI	-	No	[61]
	IONPs	Bevacizumab	^99m^Tc/ DMSA (dimercaptosuccinic acid)	SPECT/MRI	-	No	[65]
	Silica NPs	CAR T cells	^89^Zr/ chelator-free	PET/NIRF	-	No	[66]
	Silica NPs	α-melanocyte stimulating hormone	^177^Lu-DOTA	SPECT	Radionuclide therapy		[99]
	CuNCs	AS1411	^99m^Tc/ chelator-free	MRI/FI/SPECT/CT	-	No	[82]
	UCNPs	apo-human serum transferrin protein	^99m^Tc/ chelator-free	IRTI/CT/MRI/SPECT	-	No	[78]
	UCNPs	Trastuzumab	^177^Lu/ chelator-free ^68^Ga/ chelator-free	NIRF /SPECT/PET	Radionuclide therapy	Yes	[100]
	Copper sulfide NPs	Cyclic RGDfK peptide	^64^Cu/ chelator-free	PET/CT	photothermal ablation therapy	Yes	[98]
	Copper sulfide NPs	Cell Membrane:	^99m^Tc/ chelator-free	SPECT/CT/PA	PTT	Yes	[101]

#### Toxicity and challenges regarding the clinical translation of radiolabeled nanoparticles

In the past ten years, a number of radiolabeled nanoparticles have been suggested for the purpose of utilizing PET imaging to detect cancer in preclinical settings [31]. Despite the collective research endeavors conducted worldwide, with the exception of silica nanoparticles known as Cornell dots, the integration of radiolabeled nanoparticles into clinical practice has been limited thus far [142]. These challenges arise primarily due to the complexities associated with attaining desirable pharmacokinetic characteristics, ensuring consistent homogeneity of nanoparticles, and addressing problems related to radiochemical stability, toxic effects, biodegradation, and clearance. Moreover, the clinical translation of radiolabeled nanoparticles is impeded by many technological and regulatory challenges. Within this section, we have examined the primary obstacles encountered by radiolabeled nanoformulations and the distinct concerns that arise from a clinical or translational perspective.

##### Biological challenges

The biological distribution of radiolabeled nanostructures often exhibits a limited distribution inside the specific compartment in which they are delivered. When provided in a localized manner, these substances have a tendency to remain in the vicinity of the injection site and are subsequently eliminated gradually through the process of lymphatic drainage. Nevertheless, this methodology is constrained to surface-level malignancies, therefore exhibiting a restricted range of applicability. When administered orally, nanoparticles primarily reside in the gastrointestinal system and are eliminated by fecal excretion. The tumor-targeting efficacy of radiolabeled nanoparticles delivered via this technique is often modest, thereby limiting their utilization for cancer NMI. Intravenous injection is the predominant method of administering radiolabeled nanoparticles, which has gained widespread acceptance in the scientific community. Following intravenous administration, the uptake of radiolabeled nanoparticles takes place in tumors, which are usually defined by fenestrated endothelium and higher vascular permeability. This uptake is influenced by factors including charge, hydrodynamic diameter, and surface properties (e.g., the presence of a "stealth" coating like PEGylation) [143]. Additionally, sites of inflammation also exhibit uptake of these nanoparticles [144]. One significant drawback of this methodology is its limited capacity to achieve a high tumor-to-background ratio, mostly attributed to the non-specific absorption of radiolabeled nanomaterials into healthy organs like the spleen and liver.

The mononuclear phagocyte system (MPS) is comprised of a network of phagocytic cells mostly located in the spleen, liver, and lymph nodes. Upon injection, these cells, particularly macrophages, promptly sequester nanoparticles. This process leads to significant and fast absorption in these organs. The process of sequestration is initiated by the opsonization of nanoparticles, which entails the adsorption of plasma proteins onto the surface of radiolabeled nanomaterials [144]. The development of the protein coating surrounding nanoparticles is contingent upon several parameters, encompassing the size of the nanoparticles, their hydrophobic properties, surface charge, and surface chemistry. Following the process of protein adsorption, the radiolabeled nanoparticles exhibit targeted binding to receptors located on the exterior membrane of phagocytes. Following this, the nanoparticles that have been tagged with radioisotopes are taken up by cells, transferred to phagosomes, and then merged with lysosomes. Furthermore, the opsonization process frequently impacts active-targeting techniques for radiolabeled nanoparticles, as it leads to the masking of targeting ligands by attached proteins on the nanoparticle surface. Consequently, this results in a notable decrease in the specificity of the nanoparticles. Furthermore, the liver and spleen exhibit significant absorption and extended retention of nanoparticles, leading to notable toxicity concerns for specific inorganic nanoparticles, like ^64^CuS nanoparticles [144, 145]. This topic is of considerable importance and warrants attention. In order to overcome this constraint, PEG is extensively employed for the purpose of modifying the surface of nanoparticles, owing to its documented attributes of "stealth" features and biological compatibility exhibited by the PEGylated nanoparticles [143]. The prevailing belief is that PEGylation increases the duration of nanoparticle circulation and leads to a substantial decrease in opsonization. Consequently, this results in improved absorption of radiolabeled nanoparticles in tumors through both passive targeting (the EPR effect) and active targeting mechanisms. Nevertheless, a number of conflicting studies have demonstrated limited, and in some cases, adverse, outcomes associated with PEGylation. These investigations have provided evidence that the process of PEGylation leads to a notable decrease in the internalization of nanoparticles by certain cells, promotes stronger binding of the nanoparticles to proteins in the bloodstream, and initiates an immune response that assists in the swift elimination of the nanomaterials from the biological system [146]. Hence, it is postulated that the extensive dependence on PEGylation in the utilization of radiolabeled nanoparticles for cancer imaging could hinder their advancement towards clinical use.

**Fig. 7:**
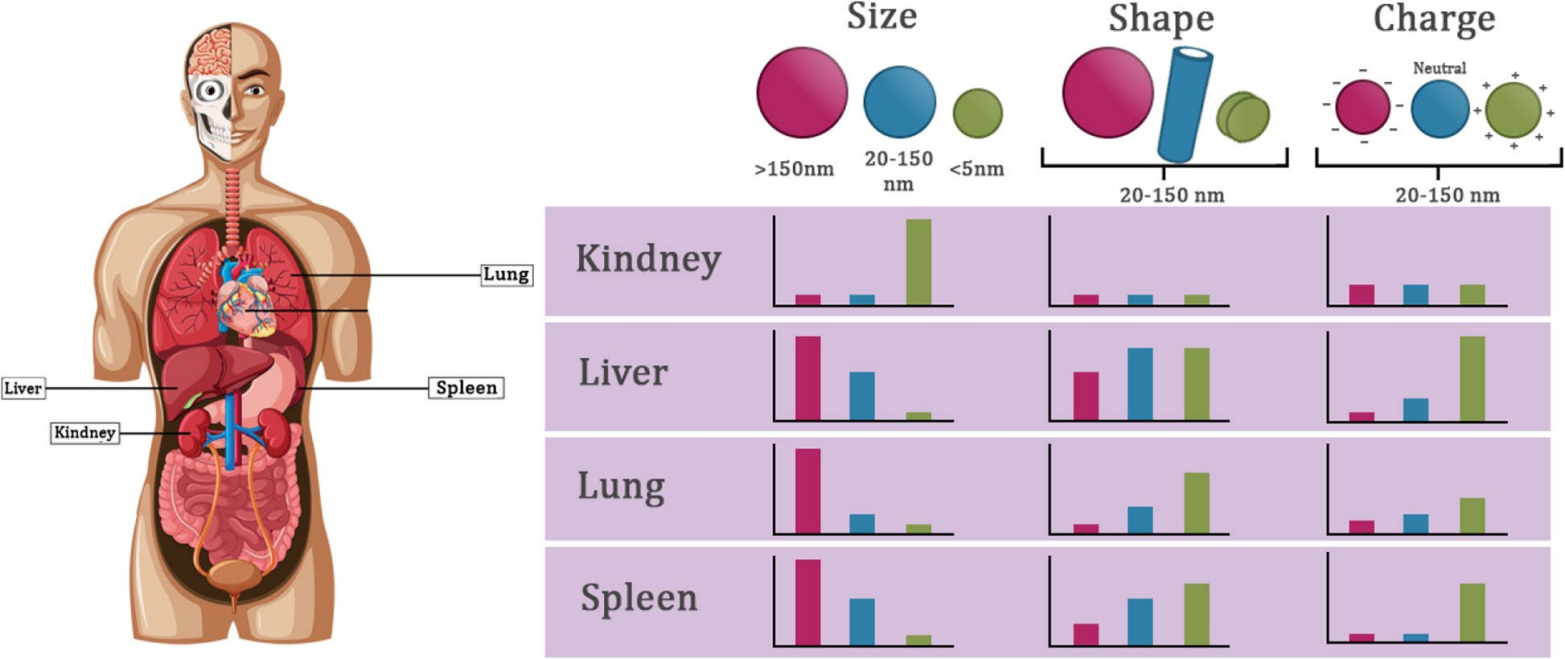
The biodistribution of radiolabeled nanoparticles is influenced by their shape, size, and surface charge. These factors play a crucial role in determining how these nanoparticles are distributed across various organs, such as the lungs, liver, kidneys, and spleen. This feature has significant importance in the context of their potential clinical applications

The biodistribution of radiolabeled nanoparticles in several organs, such as the liver, lungs, kidneys, and spleen, is influenced by factors such as their shape, size, and surface charge (see Fig. 7). The size of nanoparticles has a significant impact on several biological processes, characterized by certain size thresholds. These processes encompass the duration of nanoparticle circulation inside the body, the ability to penetrate leaky blood vessels, and the absorption of nanoparticles by macrophages. Hence, it is imperative to fabricate nanoparticles possessing exact dimensions and exhibiting great monodispersity. For instance, nanoparticles possessing hydrodynamic dimensions smaller than 5 nm demonstrate rapid renal clearance following intravenous administration, resulting in reduced toxicity in comparison to larger nanoparticles [144]. The endothelial layer in the liver has a non-continuous structure and contains vascular fenestrations that have a size range of 50–100 nm. This characteristic of the liver endothelium results in the non-specific accumulation of nanoparticles that are larger in size. Moreover, the process of splenic filtration is responsible for the retention of particles larger than 200 nm. This is mostly attributed to the dimension range of interendothelial cell slits, which typically fall between 200 and 500 nm. In general, nanoparticles with an average hydrodynamic diameter of around 100 nm exhibit prolonged circulation throughout the body. An extended circulation half-life of radiolabeled nanoparticles is expected to enhance their propensity to extravasate through fenestrations throughout tumor vasculature, which exhibit a size range of 380–780 nm. The diverse geometries of the nanoparticle manifest distinct flow properties that substantially alter the duration of circulation for radiolabeled nanoparticles, interactions with cell membranes, and absorption by macrophages, thereby impacting the dispersion of nanoparticles across several organs (see Fig. 7) [144]. The presence of surface charge on nanoparticles is a result of unique surface chemistries. This surface charge has implications for circulation half-life, opsonization, and how they interact with resident macrophages in organs that comprise the MPS (see Fig. 7) [144]. Positively charged nanoparticles have a higher susceptibility to sequestration by macrophages throughout the liver, lungs, and spleen. Conversely, nanoparticles with a neutral or slightly negative charge have extended circulatory half-lives, resulting in reduced accumulation within the MPS organs [144].

The field of tumor targeting based on nanoparticles has been significantly influenced by the EPR effect in tumors [147]. However, it has been observed that the magnitude of this phenomenon varies dramatically depending on the level of tumor vascularity. Moreover, the EPR effect is often observed by the absorption of radiolabeled nanoparticles in the vasculature of tumors. However, it is important to note that interstitial fluid pressures may still prevent the movement of nanoparticles into specific target areas. Moreover, it is important to note that while the EPR effect has been extensively demonstrated in preclinical research using small animal models, there is a lack of comparable data in clinical trials involving people. The clinical translation of radiolabeled nanoparticles is hindered by this significant challenge [144, 148]. A further concern pertaining to disparities between human and animal trials is the optimization of the quantity of targeting ligands present on a nanoparticle. In general, there may be a lack of correlation observed between numerical values in tiny animals and people, as well as between two individuals within the human population [144]. Furthermore, it is worth noting that in the majority of preclinical investigations, the in vitro radiochemical stability assessments of radiolabeled nanoparticles are conducted in a manner that lacks consideration for the actual biological environments, which would typically contain appropriate concentrations of biological chelators.

One further significant concern pertaining to nanoparticles involves the assessment of their biological destiny and enduring biocompatibility. Despite several preclinical investigations that have shown biodistribution, cellular interactions, and elimination of radiolabeled nanoparticles, the lack of standardization in experimental circumstances prevented the normalization of these findings [148]. Hence, the overall findings of this research were unable to be substantiated, even within the preclinical domain. Recent results indicate that the biodegradability and biocompatibility of nanoparticles when delivered in vivo are influenced by several design characteristics, such as size, shape, charge, and porosity [144, 148]. Consequently, it is imperative to conduct analytical evaluations of these parameters at both the preclinical and clinical stages, as applicable, in order to acquire essential data regarding the toxicity, biodistribution, and biological compatibility of radiolabeled nanoparticles. This is crucial for optimizing their potential for success in clinical applications. Moreover, it is imperative to conduct comprehensive dosimetry investigations prior to the administration of therapeutically significant quantities of radiolabeled nanoparticles in human subjects, specifically for the purpose of PET imaging in cancer patients. In order to enhance the accuracy and reliability of dosimetry investigations, it is imperative to incorporate comprehensive kinetic modeling and distribution analyses that elucidate the impact of the physicochemical and biological characteristics of radiolabeled nanoparticles.

##### Regulatory and technological challenges

In order to guarantee the clinical effectiveness of future radiolabeled nanoparticle formulations, it is crucial to implement various methodologies that tackle the significant technological challenges associated with these nanoparticles. These challenges include process optimization, scaled-up synthesis, quality control, efficiency predictions, and quality assurance. Addressing these challenges through appropriate methods is imperative. In addition, it is imperative to adhere to current good manufacturing procedure (cGMP) compliance during the creation of radiolabeled nanoparticles for human use. This is necessary to verify that the quality of the finished product aligns with the specified acceptance criteria. The standards governing the manufacturing of molecular imaging agents in accordance with current cGMP, as specified in the Code of Federal Standards, have been authorized by the United States Food and Drug Administration (US-FDA). The primary objective of enforcing cGMP is to prevent patients from being exposed to potential risks arising from insufficient quality and safety measures. Additionally, it aims to promote uniformity in the implementation of regulatory standards. Any departure from the authorized procedure of preparation would necessitate substantial validation prior to the utilization of the treatment on patients. The sterile compounding requirements outlined in the United States Pharmacopeia (USP) < 797 > provide a set of minimum practices and quality standards that are legally binding. These standards are designed to govern the creation of compounded sterile medicinal products, taking into account the most up-to-date scientific knowledge and adhering to the best practices in sterile compounding [149].

The pursuit of cGMP compliance in the manufacturing of radiolabeled nanoparticles is an appealing concept, yet it presents significant challenges. These challenges encompass the need for highly skilled personnel, the utilization of controlled substances and procedures, the availability of appropriate equipment, the synthesis and radiolabeling of nanoparticles within designated clean environments, the implementation of validated processes and analytical techniques, comprehensive documentation of the entire process, registering the usage of the radiolabeled agent with national or regional health authorities, and the authorization for human use by qualified individuals. In preclinical settings, the production and radiolabeling of nanoparticles are often performed using manual procedures. Nevertheless, the implementation of this methodology for extensive clinical applications presents significant challenges. Hence, it is advisable to contemplate the utilization of automated synthesis modules owing to the subsequent benefits: 1) provision of resilient and replicable synthesis of the radiolabeled nanoparticles; 2) mitigation of operator involvement, thereby minimizing operational inaccuracies; 3) assurance of radiation safety; 4) facilitation of compliance with cGMP and provision of comprehensive traceability of the process, a crucial aspect given the substantial regulatory obligations; and 5) mitigation of the likelihood of bacterial contamination of the radiolabeled agent, a significant consideration in the context of clinical application. It is crucial to acknowledge that, despite the commendable qualities of an automation strategy, thorough examination and justification of each operational step's requirements are imperative throughout the implementation of the automation process. This is because every step directly influences the entire cost of the module. In addition, the automation process presents the issue of adapting the module to accommodate new procedures that are based on emerging nanoplatforms or novel radioisotopes. This adaptation must be achieved while ensuring complete automation and adherence to the rules set out by cGMP. However, for optimal efficacy in overcoming specific regulatory obstacles, it is imperative to tailor automated synthesis modules to conform with the unique legislative, regulatory, and institutional frameworks of the local context.

## Conclusion and perspectives

The concept of employing numerous modalities during a single imaging session arises from the observation that modalities characterized by high sensitivity tend to exhibit suboptimal resolution, whereas those with high resolution often have diminished sensitivity [150]. The advent of hybrid technologies for imaging has spurred significant endeavors in the creation of probes aimed at enhancing the advantages of hybrid instrument technology [13, 151]. Consequently, a diverse range of nanoparticle formulations have been created to facilitate the non-invasive visualization of tumors and capitalize on the exploitable attributes specific to cancer [152].

Despite the considerable increase in the utilization of radiolabeled nanoparticles for multimodality tumor imaging in recent years, the majority of investigations conducted so far have been primarily focused on establishing the feasibility of this approach. Consequently, more research endeavors are imperative to facilitate the transition of radiolabeled nanoparticles from the experimental stage to clinical implementation in the foreseeable future. There are a number of crucial concerns that deserve attention in relation to the advancement of radiolabeled nanoparticles. The primary obstacle encountered in in vivo imaging is the absorption of radiolabeled nanoparticles by the reticuloendothelial system (RES) organs, including the liver and spleen. The physicochemical characteristics, including dimensions, adaptability, hydrophobicity, and surface charge, of nanoparticles have a significant impact on their distribution throughout an organism and their removal from the body. Nanoparticles that possess a rigid and globular shape and have a size smaller than 6 nm have the capability to be eliminated via the renal pathway. Specifically, nanoparticles ranging from 4–8 nm in size are promptly cleared from the bloodstream by the reticuloendothelial system, resulting in fast accumulation inside the spleen and liver and subsequent elimination via the hepatobiliary pathway [153]. Therefore, it may be inferred that nanoparticles with smaller sizes will exhibit less absorption of RES, leading to improved imaging characteristics. Furthermore, the separation of the radionuclide (usually in the form of a metallic ion) from the chelator or the detachment of the radionuclide-containing polymeric coating from the nanoparticle might result in substantial accumulation in healthy organs and diminish the particularity of the radiolabeled nanoparticles. The consideration of a radionuclide-chelator combination with improved in vivo stability is necessary. It is often necessary to conduct meticulous in vitro tests to assess the durability of radiolabeled nanoparticles before proceeding with in vivo research. Furthermore, in order to enhance the selective binding of radiolabeled nanoparticles to the tumor site, careful selection of targeting moieties, including peptides and antibodies, is necessary [154]. Additionally, a thorough investigation of the physicochemical features of nanoparticles, including size and surface charges, is crucial. In addition, it is imperative to give due consideration to the cellular toxicity of nanoparticles while designing radiolabeled nanoparticles for the purpose of multimodality tumor imaging. Nanoparticles exhibiting significant cellular toxicity are unlikely to be considered for therapeutic investigation. Certain inorganic nanoparticles composed of metallic elements, like gadolinium and cadmium (e.g., quantum dots), have been identified as having hazardous properties [155]. Certain carbon-based nanoparticles also exhibit biological toxicity [156]. Prior to the administration of nanoparticles for tumor diagnostics in humans, it is imperative to conduct thorough investigations into the possible toxicity of these nanoparticles. Generally, there is a greater preference for biocompatible materials, particularly those derived from the human body, in comparison to heavy metals when considering their suitability for human applications.

As previously stated, the process of developing radiolabeled nanoparticles is not straightforward. The utilization of sophisticated chemical approaches is expected to maintain its significance in the conceptualization and advancement of radiolabeled nanoparticles for the purpose of multimodality tumor imaging. One illustrative instance is the utilization of "click chemistry," [157] which provides a framework for the implementation of broad, adaptable, and efficient synthetic conversions in order to fabricate a wide array of molecules with high yields. The modular nature of click chemistry enables the precise adjustment of the pharmacokinetic and pharmacodynamic characteristics of imaging probes. Click chemistry has demonstrated its superiority in meeting several requirements, such as selectivity, biocompatibility, yield, and stereospecificity. Significant advancements have been achieved in the catalyst-free variation of azide-alkyne click chemistry due to recent developments in this field. The activation energy for the cycloaddition process is reduced by the release of ring strain within a cycloalkyne precursor, enabling the ligation to be carried out with excellent effectiveness in the absence of a catalyst [158, 159]. The utilization of click chemistry is anticipated to increasingly emerge as a conventional approach in the foreseeable future, including a diverse array of applications pertaining to the fabrication of radiolabeled nanoparticles.

It is noteworthy to emphasize that radiolabeled nanoparticles have the potential to be produced and utilized not only for the purpose of tumor diagnostics but also as therapeutic agents, commonly referred to as "theranostics" [160]. The field of nanomedicine has made significant advancements in utilizing tumor-targeted nanoparticles for the purpose of delivering radionuclides in a precise way. This approach aims to enhance the effectiveness and safety of cancer imaging and therapy. The utilization of radiolabeled nanoparticles following their conjugation with suitable chemotherapeutic agents enables the acquisition of imaging data and facilitates the monitoring of delivery kinetics, tumors, and medication effectiveness. This approach holds promise for enhancing treatment regimens by improving therapeutic efficacy while minimizing harm to healthy tissues. It is foreseeable that theranostic medicines based on radiolabeled nanoparticles will assume significant roles in the forthcoming years in the domains of cancer detection, early-stage anti-cancer medication development, and the formulation of cancer therapy plans.

In conclusion, it is imperative to devise innovative ways that may be employed to ascertain cancer with heightened sensitivity and enhanced predictive efficacy. The utilization of radiolabeled nanoparticles has demonstrated significant potential as molecular probes for the purpose of multimodal tumor imaging. It is anticipated that the utilization of quantitative multimodality imaging, employing newly developed radiolabeled nanoparticles, would enable the exact and accurate evaluation of biological indicators in cancer in a timely way. Consequently, this advancement will facilitate the progression towards customized cancer medication.

## Data Availability

Not applicable.

## Abbreviations

COVID-19: Coronavirus disease 2019
MRI: Magnetic resonance imaging
CT: Computed tomography
SPECT: Single-photon emission computed tomography
PET: Positron emission tomography
EPR: Enhanced permeability and retention
RPT: Radiopharmaceutical therapy
PSMA: Prostate-specific membrane antigen
GRP: Gastrin-releasing peptide
DOTA: 1,4,7,10-Tetraazacyclododecane-1,4,7,10-tetraacetic acid
HDL: High-density lipoprotein nanoparticle
^18^F‐FDG: 2-Deoxy-2-[^18^F] fluoro-D-glucose
HER2: Human epidermal growth factor 2
SIONPs: Superparamagnetic iron oxide nanoparticles
TEx: Tumor cell-derived exosomes
NIRF: Near-infrared fluorescence
QDs: Quantum dots
UCNPs: Upconversion nanoparticles
DSPE-PEG-FA: 1,2-Distearoyl-sn-glycero-3-phosphoethanolamine-N-[folate(polyethylene glycol)-2000]
CuNCs: Copper nanoclusters
PEG: Polyethylene glycol
HPAO: 3-(4-Hydroxyphenyl) propionic acid-OSu
PTT: Photothermal therapy
PDT: Photodynamic therapy
MPS: Mononuclear phagocyte system
cGMP: Current good manufacturing procedure
US-FDA: United States Food and Drug Administration
USP: United States Pharmacopeia
RES: Reticuloendothelial system
SERS: Surface-enhanced Raman scattering
CuS-Fn NCs: CuS-ferritin nanocages
PAI: Photoacoustic imaging
CNPs: Crosslinked nanoparticles
MNPs: Melanin nanoparticles
CL: Cherenkov luminescence
CRET: Cerenkov resonance energy transfer
LSPR: Localized surface plasmon resonance
ID/g: Injected dose per gram
GSH: Glutathione

## Data availability

No datasets were generated or analysed during the current study.
